# Construction of a T7 phage display nanobody library for bio-panning and identification of chicken dendritic cell-specific binding nanobodies

**DOI:** 10.1038/s41598-022-16378-x

**Published:** 2022-07-15

**Authors:** Hai Xu, Ling Li, Bihua Deng, Weiming Hong, Ruiting Li, Zijie Guo, Jibo Hou, Roshini Govinden, Hafizah Y. Chenia

**Affiliations:** 1 Jiangsu Key Laboratory for High-Tech Research and Development of Veterinary Biopharmaceuticals, Jiangsu Agri-Animal Husbandry Vocational College, Taizhou, 225300 Jiangsu Province People’s Republic of China; 2 Discipline: Microbiology, School of Life Sciences, College of Agriculture, Engineering and Science, University of KwaZulu-Natal, Durban, 4000 South Africa; 3 Institute of Veterinary Immunology and Engineering, Jiangsu Academy of Agricultural Science, Nanjing, 210014 Jiangsu Province People’s Republic of China; 4 Jiangsu Co-Innovation Center for Prevention and Control of Important Animal Infectious Diseases and Zoonoses, Yangzhou, 225009 People’s Republic of China

**Keywords:** Biotechnology, Nanobiotechnology, Vaccines

## Abstract

Dendritic cells (DCs) are the antigen-presenting cells that initiate and direct adaptive immune responses, and thus are critically important in vaccine design. Although DC-targeting vaccines have attracted attention, relevant studies on chicken are rare. A high diversity T7 phage display nanobody library was constructed for bio-panning of intact chicken bone marrow DCs to find DC-specific binding nanobodies. After three rounds of screening, 46 unique sequence phage clones were identified from 125 randomly selected phage clones. Several DC-binding phage clones were selected using the specificity assay. Phage-54, -74, -16 and -121 bound not only with chicken DCs, but also with duck and goose DCs. In vitro, confocal microscopy observation demonstrated that phage-54 and phage-74 efficiently adsorbed onto DCs within 15 min compared to T7-wt. The pull-down assay, however, did not detect any of the previously reported proteins for chicken DCs that could have interacted with the nanobodies displayed on phage-54 and phage-74. Nonetheless, Specified pathogen-free chickens immunized with phage-54 and phage-74 displayed higher levels of anti-p10 antibody than the T7-wt, indicating enhanced antibody production by nanobody mediated-DC targeting. Therefore, this study identified two avian (chicken, duck and goose) DC-specific binding nanobodies, which may be used for the development of DC-targeting vaccines.

## Introduction

China is among the top countries in poultry meat and egg production and consumption^1^. Disease control is the key element for improving poultry production and food safety and, thus, human well-being^2^. The first line of infectious disease control is to establish and maintain immunity, thus a vaccine is considered as one of the most helpful immune interventions due to its ability to induce protective immunity through the targeted direction of the immune system^3^. Various types of vaccines have been researched, produced and widely used for poultry, including inactivated vaccines, attenuated vaccines, and genetically engineered vaccines^4^. However, vaccine breakthrough infections still occur, predominantly due to inadequate quantity and poor quality of antigens in the vaccine^5^. Thus, new and improved vaccines for poultry diseases are urgently required.

Generally, an organism’s defense system consists of both specific and nonspecific immune responses towards invading pathogens. Specific (adaptive) immunity, including response to vaccines, is primarily elicited by dendritic cells (DCs), specialized leukocytes adapted for antigen capture, processing and presentation to naive T cells to initiate primary immune responses^6,7^. Because of the unique capacity mentioned above, DCs are usually utilized as the antigen target for the induction of efficient and strong immune responses^8^. Consequently, to pursue more effective candidates for treatment and prevention of disease, DC-targeting strategies have been proposed, and hundreds of DC-targeting studies have been published^9–11^. There are several approaches to achieve DC-targeting, i.e., autologous DCs are loaded with antigens ex vivo and re-injected into hosts^12,13^, or DCs can be targeted in situ by conjugating the antigen with DC receptor-specific monoclonal antibodies or ligands^14,15^. The widely used approach for preventative vaccine development, involves selective targeting of DC-specific endocytic receptors by linking the relevant antigen to antibodies or ligands^9^. Moreover, the immune response initiated by the DCs tends to depend upon the context in which the antigen was captured^8^. Therefore, exploration of new DCs targeting antibodies or ligands is significant for the enhancement and development of chicken vaccines.

The immune systems of chicken and mammals are similar, but there are remarkable differences associated with immunological cells, genes, lymphoid tissue and variation in antibodies^16^. Many immunity genes such as those coding for chemokines, chemokine receptors, and Toll-like receptors vary between chicken and mammals^17,18^. The chicken lacks lymph nodes, but has the bursa of Fabricius and cecal tonsils, which are absent in mammals. However, chicken DCs do have similar functions to those in mammals, although the most responsive avian antigen-presenting cells (APCs) are still unknown^19^. Due to the lack of research on systematic immunity in chickens compared with mouse, human and swine, the development of a chicken DC targeting vaccine is lagging.

Phage display technology is an in vitro selection platform that is used to identify specific polypeptide chains displayed on the surface of bacteriophage by virtue of their affinity adsorption^20^. The main cornerstone of phage display technology is the application of bacteriophages to display proteins by using different bacteriophage systems such as the T4, T7, and lambda, but the M13 phage is still a classic example of a non-lytic filamentous phage^21^. Phages have been an important tool in the bioengineering field, and are exploited for a range of applications including ligand identification^22^, drug research and development^23^, biosensors^24,25^ and vaccine development^26^. Larger-sized molecules like antibodies have been coupled with phage display technology, leading to the advancement of phage display technology application in fields like APC targeting delivery^27^.

**Figure 1:**
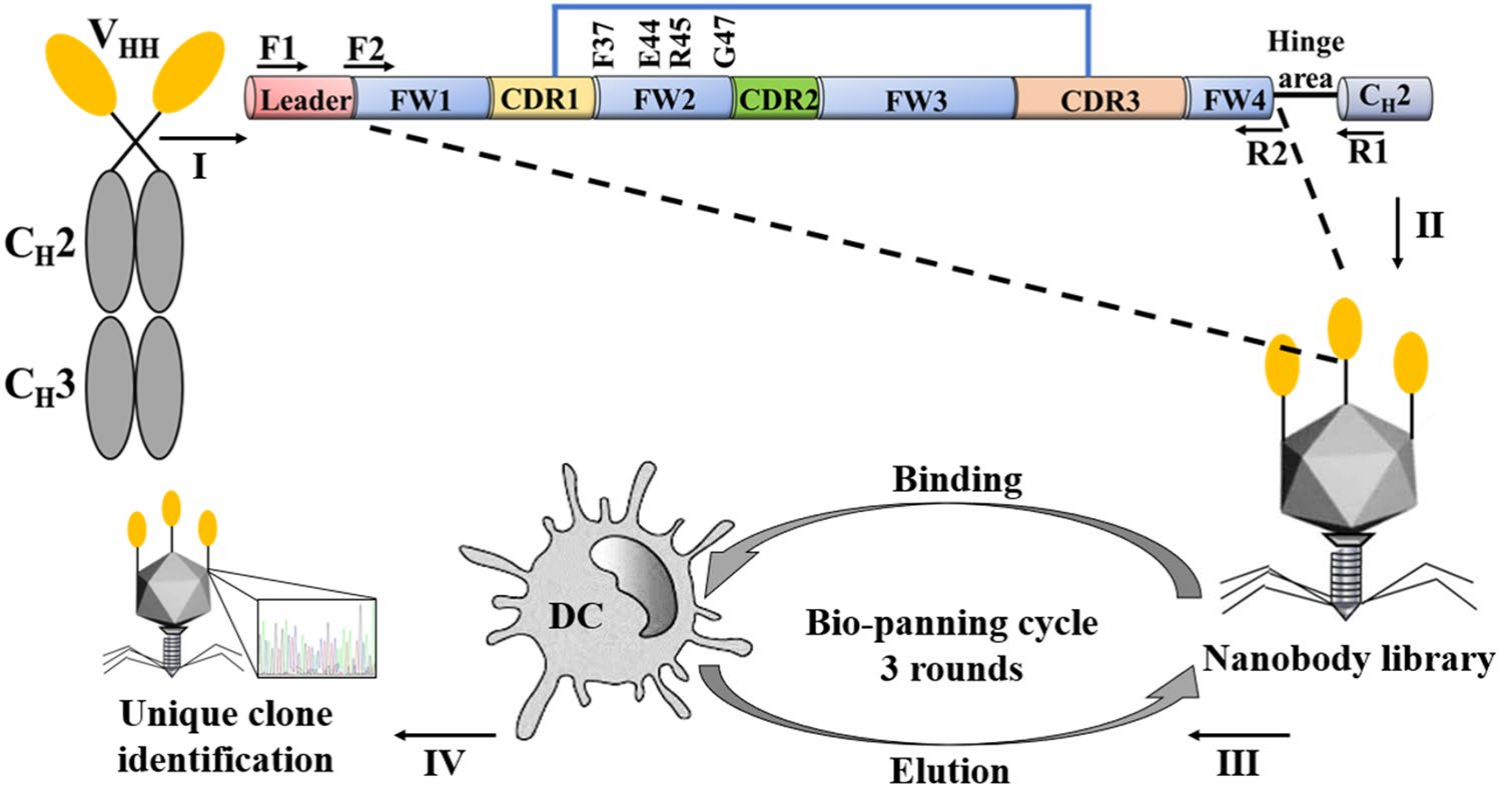
Schematic of T7 phage display nanobody library construction and bio-panning. Schematic of strategies used to construct the nanobody library, bio-panning of dendritic cell (DC)-specific binding VHHs, and sequence analysis of single phage plaque clones. (I). Molecular structure of nanobody. (II). The nanobody VHH genes were inserted into the T7 phage genome to rescue the nanobody library. (III). The bio-panning cycles of chicken DCs. (IV). Sequence analysis of a single phage clone.

Considering the difference between mammals and chickens, mammalian ligands to DCs have some limitations when applied in chickens^10^. To select chicken DC-targeting ligands, bio-panning was implemented in the T7 phage display heavy chain variable region (VHH, nanobody) gene library on chicken bone marrow-derived immature DCs in this study (Fig. 1). First, bio-panning was used to discover high affinity nanobodies that could bind specifically to chicken DCs. Then, the potential application of the selected nanobodies as hapten carriers in promoting antibody formation was verified.

## Results

### Construction of a high diversity nanobody library

The cDNA was prepared using the extracted total RNA from alpaca peripheral blood lymphocytes by reverse transcription (RT). The VHH genes were amplified to produce amplicons of about 450 bp by stepwise PCR using the synthesized cDNA as a template. The primary VHH library was generated by cloning the VHH gene repertoire into the T7 select 415-1b vector, followed by in vitro packaging. Subsequently the primary library generated was amplified by the liquid lysate method. The titers of the primary and amplified library were 2.73 × 10^9^ PFU/mL and 1.65 × 10^11^ PFU/mL, respectively. Diversity analysis of the primary library was carried out by PCR detection of 20 random phage clones (Supplementary Fig. S1-A). Sequence analysis indicated the typical nanobody structure with the framework region (FR) and complementarity determining region (CDR). In addition, differences in amino acid sequences of the CDRs indicated a high diversity library (Supplementary Fig. S2). Nanobodies displayed on the T7 phage surface were detected by Western-blot (Supplementary Fig. S1-B). Four kinds of T7 phage capsid protein had been identified in lane 2, including p10B-VHH fusion protein (51 kDa), p10B protein (36 kDa) which was expressed by the *E. coli* BL5405 host, and two truncated p10B-VHH fusion proteins with molecular weights between 51 and 36 kDa. These results indicated that the T7-VHH library has been successfully constructed and could be used for bio-panning purpose.

### Preparation of chicken bone marrow dendritic cells

**Figure 2:**
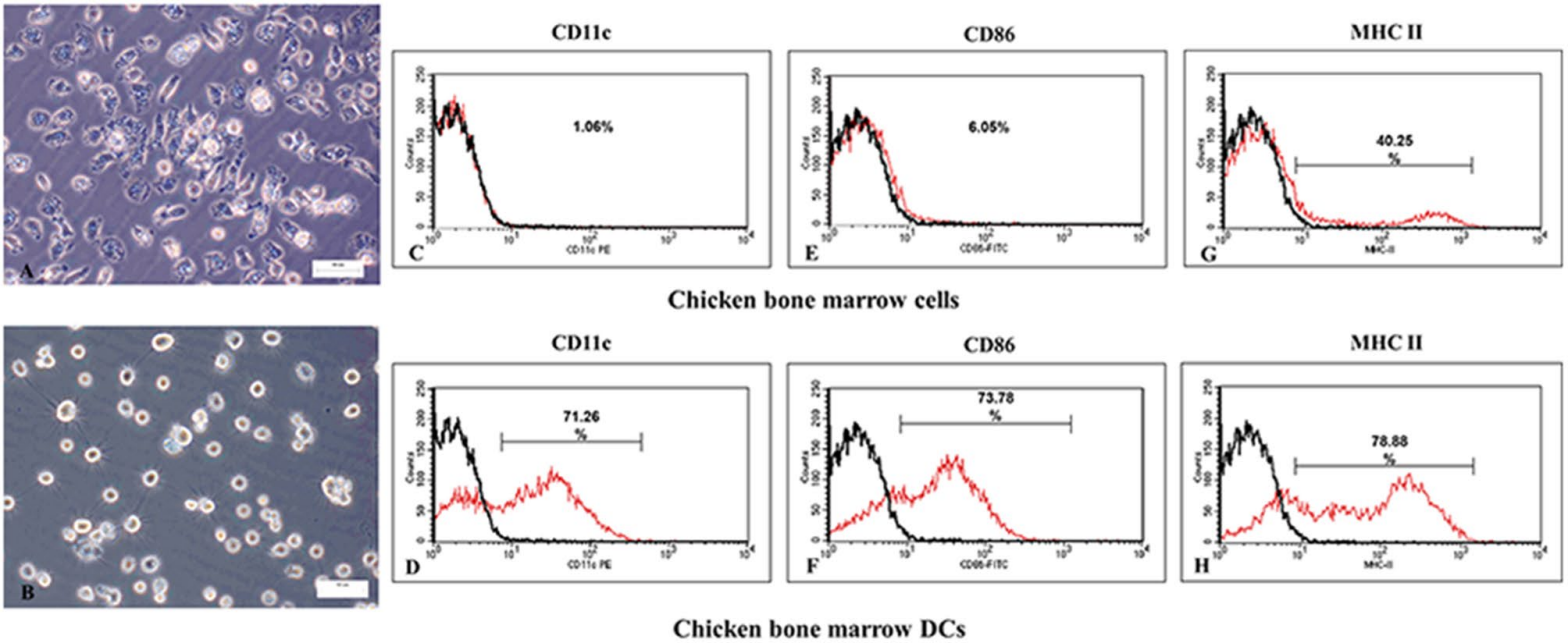
Preparation and identification of chicken bone marrow dendritic cells. (**A**) Morphology of adherent of chicken dendritic cells (DCs) at day 6. During the inducing differentiation process, the circular monocytes gradually adhered to the plate and formed cell clusters. The adherent DCs were fusiform in shape or polymorphic with obvious burr-like protrusions on the cell surface. (**B**) Morphology of DCs digested by trypsin at day 6. Polymorphic DCs switched to a spherical shape after digestion with trypsin, and the protrusions on the cell surface presented a needle-like form which varied in length. (**C**–**H**) Surface marker detection of chicken dendritic cells by flow cytometry. CD11c, CD86 and MHC II expression on the surface of chicken bone marrow-derived dendritic cells (DCs) cultured for 6 d were detected by fluorescence-activated cell sorting. Expression of the three surface markers was up-regulated in the chicken bone marrow-derived DCs after granulocyte macrophage-colony stimulating factor and interleukin-4 induction compared to chicken bone marrow cells. Isotype matched controls (black line) and the test markers (red line) are shown.

The morphology of DCs induced by GM-CSF and IL-4 was observed by inverted microscopy. During the inducing differentiation process, the monocytes adhered to the plate gradually and formed small cell clusters. Compared to the initial monocytes, the adherent cells were fusiform in shape or polymorphic with obvious burr-like protrusions on the cell surface (Fig. 2A). After trypsinization, there was a switch in the shape of DCs from polymorphic to spherical, while the protrusions on the cell surface presented a needle-like form which varied in length (Fig. 2B).

The DC surface markers CD11c, CD80 and MHC-II were analyzed at day 6 using fluorescence-activated cell sorting (FACS), and expression levels of 71.26%, 73.78% and 78.88%, respectively, were achieved (Fig. 2D, F, and H). This contrasted with the background chicken bone marrow cell expression levels of 1.06%, 6.05% and 40.25% for CD11c, CD80 and MHC-II (Fig. 2C, E and G), respectively. Both the typical cell morphology and the characteristic high density surface molecular markers were indicative of the successful differentiation of DCs and could be used for bio-panning.

### Bio-panning and characterization of DC-specific binding nanobodies

**Figure 3:**
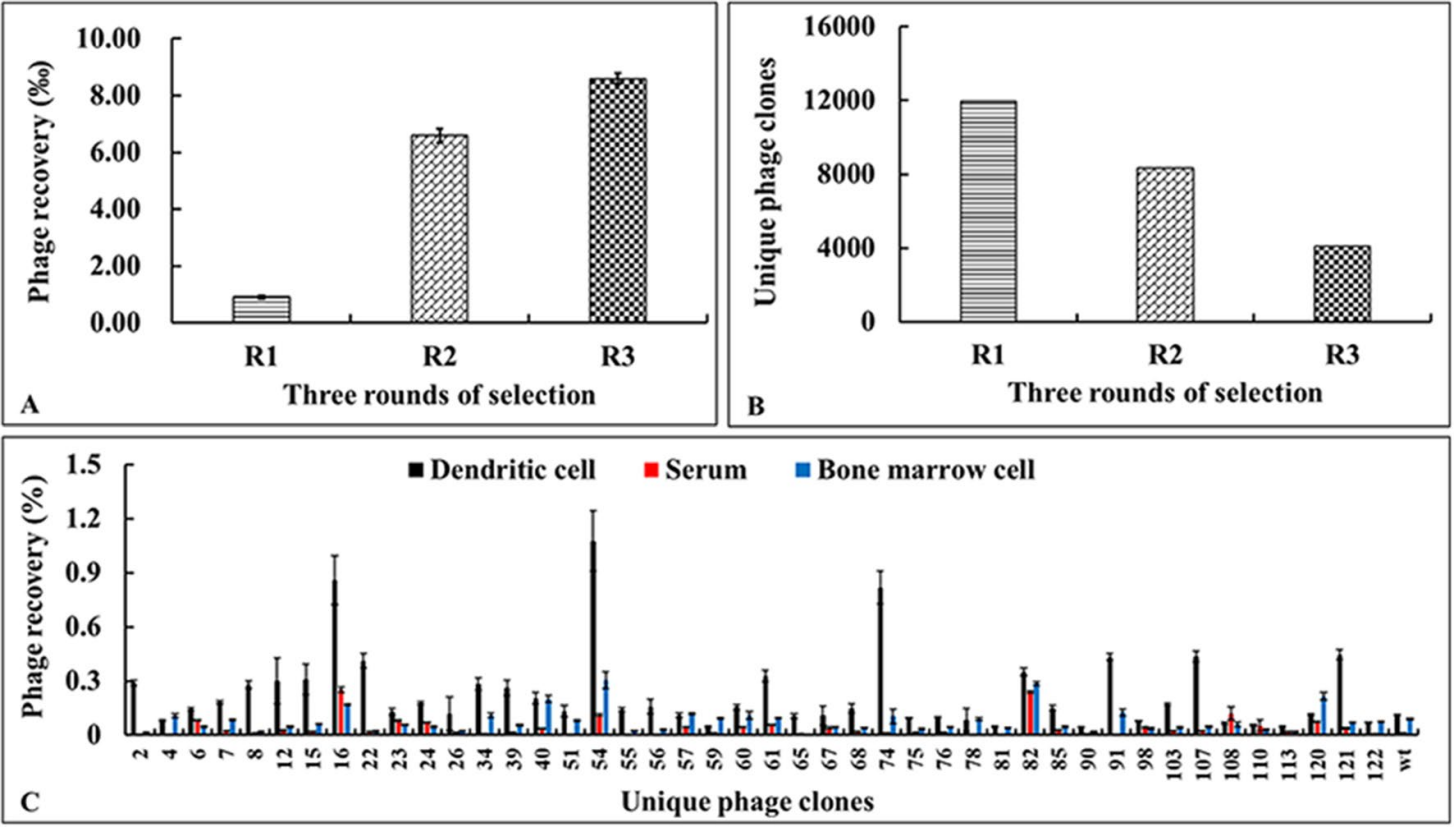
Phage recovery measurement and unique phage clone analysis of the eluates of each round of bio-panning. (**A**) Phage recovery was calculated as the ratio of recovered phage versus the input phage as follows: phage yields (%) = (output phage/input phage) × 100. (**B**) The VHH gene in the eluate of each round of bio-panning was amplified and the unique VHH gene sequence analyzed by next-generation sequencing. (**C**) Specificity assay of 46 unique sequence phage clones. The specificity of unique sequence phage (black) towards chicken dendritic cell in comparison with chicken bone marrow cell (blue) and serum (red) is indicated. Specificity of the phages was estimated as their recovery (%) = (output phage/input phage) × 100.

To screen nanobodies that specifically bind with DCs, three rounds of phage display bio-panning were performed, and bone marrow cells were added for depletion to reduce the possibility of non-specific binding. As shown in Fig. 3A, the ratio of phage recovery in each round increased, which was considered evidence of effective screening. However, when the next generation sequencing data was assessed, it was observed that the number of phage clones with unique sequences in each round of the recovered phage had decreased (Fig. 3B), which indicated an accumulation of specific DC-binding phages during the bio-panning process.

**Figure 4:**
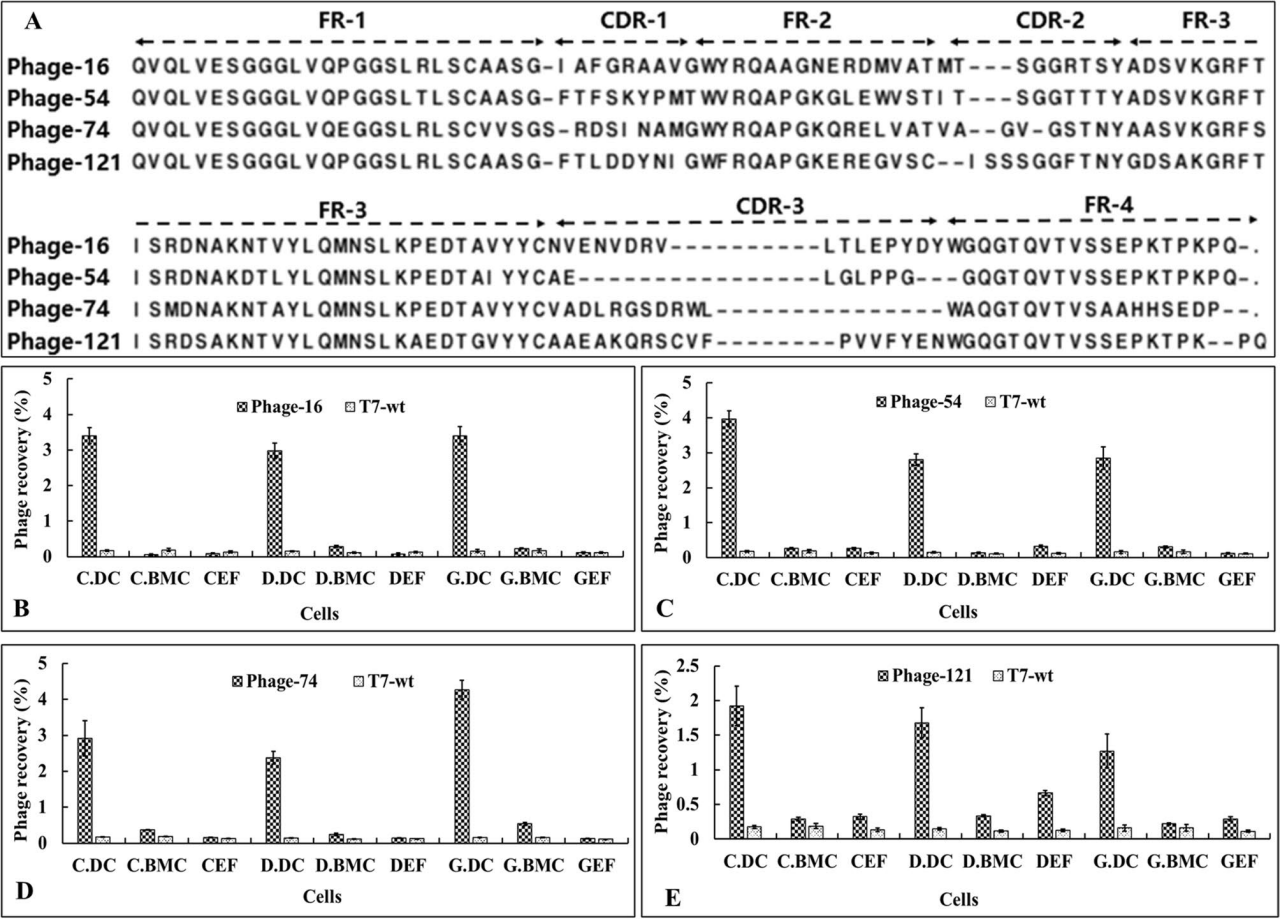
Selectivity of four phages toward different avian cells. (**A**) Phage-16, phage-54, phage-74 and phage-121 were identified from the specificity assay, and the amino sequence of surface displayed nanobody is depicted with four frameworks (FW1-FW4) and three complementary determining regions (CDR1-CDR3). (**B**–**E**) Binding of four phages to the target cell lines, chicken bone marrow derived dendritic cells (C.DC), compared to other avian cells derived from chicken, duck and goose (C.BMC (chicken bone marrow cells), CEF (chicken embryo fibroblast), D.DC (duck dendritic cells), D.BMC (duck bone marrow cells), DEF (duck embryo fibroblast), G.DC (goose dendritic cells), G.BMC (goose bone marrow cells), GEF (goose embryo fibroblast)). T7-wt phage devoid of nanobody display was used as a negative control for each assay. Phage recovery was calculated as the ratio of recovered phage versus the input phage as follows: phage recovery (%) = (output phage/input phage) × 100.

After three rounds of screening, 125 phage clones were randomly selected for sequencing, and 46 phage clones with unique CDR sequences were identified (Supplementary Fig. S3). To further characterize the selected phage clones, the specificity, i.e., the ability of a phage probe to associate with its target due to the presence of a specific nanobody displayed on the surface, and the selectivity, i.e., the ability of a phage probe to discriminate its cognate target from a mixture of targets were determined. The specificity of 46 unique sequence phage clones was evaluated and is summarized in Fig. 3C. Twelve phage clones with strong affinity and specificity (phage recovery higher than 0.3%) towards chicken DCs were identified. The intact amino sequence of the VHH display on phages-16, 54, 74 and 121 were analyzed in Fig. 4A, and the amino composition of the CDRs was different between these four clones. Further, the selectivity of the four phage clones was evaluated (Fig. 4B–E). It was observed that phages-54, -74, -16 and -121 bound not only to chicken DCs but also to duck and goose DCs, however, they barely bound to the bone marrow cells, chicken embryo fibroblasts (CEF), duck embryo fibroblasts (DEF) and goose embryo fibroblasts (GEF). These results suggest that these four phages may have great specificity for binding to DCs.

**Figure 5:**
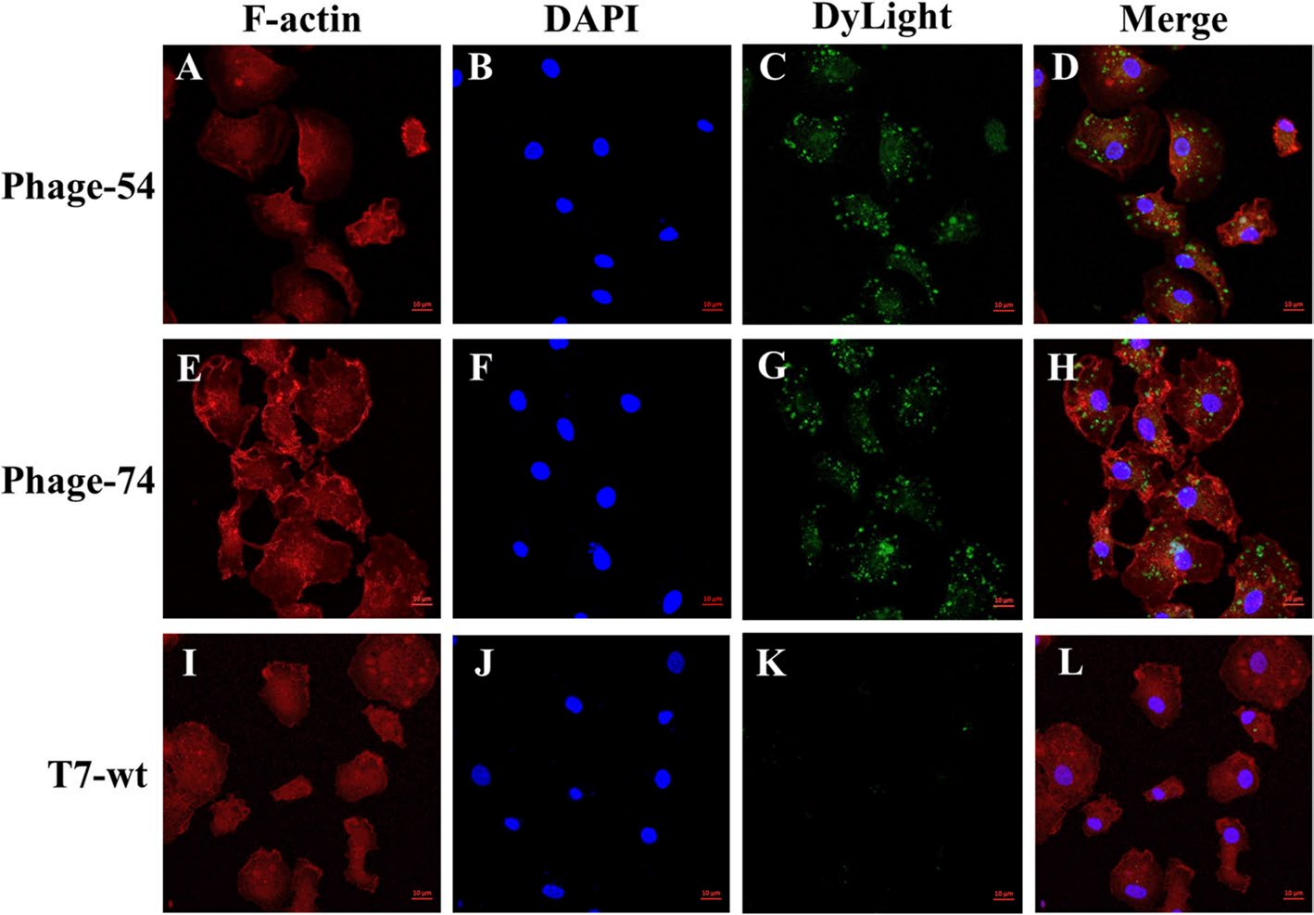
The location of phage-54 and phage-74 binding to DCs was analyzed by laser confocal microscopy. T7 phages were revealed using a Dylight Anti-T7 tag antibody as shown in green (**C**, **G** and **K**), actin was identified using the Phalloidin-iFluor 594 conjugate as shown in red (**A**, **E**, and **I**) and nuclei were identified by DAPI staining as shown in blue (**B**, **F** and **J**). T7-wt phage devoid of nanobodies did not interact with the DCs under the same conditions.

For further verification, nanobody binding to chicken bone marrow DCs was assessed by fluorescent microscopy (Fig. 5). No green fluorescence signal was evident in Fig. 5K which indicated that the T7-wt barely bound to the DCs. In contrast, phage-54 and phage-74 could adsorb to the DCs surface efficiently, as manifested by the strong green fluorescence spot covering the DC surface (Fig. 5C and -G). In addition, the suspected DC proteins that could interact with VHH-54 and VHH-74 were analyzed by pull-down and HPLC–MS assays (Table 1). The fusion proteins GST-VHH-54 and GST-VHH-74 were expressed and purified (Supplementary Fig. S4) for interaction with the DC lysate. HPLC–MS results revealed several intercellular proteins such as proteases and a couple of uncharacterized proteins, unfortunately, with almost no surface proteins being discovered (Table 1). The voltage-dependent anion-selective channel protein which was confirmed on the plasma membrane most likely interacts with VHH-74.

**Table 1:** List of nanobody-binding proteins identified by LC–MS.

Accession	Description	Score	Mass (kDa)	Matches-sig	Sequences-sig	pI	emPAI
**(a) List of VVH54-binding proteins from DCs identified by LC–MS**							
A0A1D5PV06	Protein disulfide-isomerase OS = *Gallus gallus* OX = 9031 GN = P4HB PE = 3 SV = 2	73	57.8	3	2	4.69	0.18
Q5ZLW0	Uncharacterized protein OS = *Gallus gallus* OX = 9031 GN = LCP1 PE = 2 SV = 1	66	70.2	4	4	5.16	0.2
A0A1D5NTV7	Uncharacterized protein OS = *Gallus gallus* OX = 9031 GN = ZBTB26 PE = 4 SV = 1	31	50.8	1	1	6.11	0.06
E1BZ71	THUMP domain-containing protein OS = *Gallus gallus* OX = 9031 GN = THUMPD2 PE = 3 SV = 4	30	46.1	1	1	5.32	0.07
Q08392	Glutathione S-transferase OS = *Gallus gallus* OX = 9031 PE = 2 SV = 1	30	25.3	1	1	8.86	0.13
Q1PRL4	E3 ubiquitin-protein ligase TRIM71 OS = *Gallus gallus* OX = 9031 GN = TRIM71 PE = 2 SV = 1	27	95.7	1	1	7.74	0.03
A0A1D5P0U5	Calreticulin OS = *Gallus gallus* OX = 9031 GN = CALR PE = 3 SV = 3	26	48.3	1	1	4.38	0.07
A0A1D5NYT7	BTB domain-containing protein OS = *Gallus gallus* OX = 9031 GN = KLHL26 PE = 4 SV = 2	26	68.8	1	1	5.39	0.05
F1P266	Uncharacterized protein OS = *Gallus gallus* OX = 9031 GN = ERCC5 PE = 3 SV = 2	26	127.6	1	1	4.99	0.03
A0A1D5PNH9	SET domain-containing protein OS = *Gallus gallus* OX = 9031 GN = PRDM11 PE = 4 SV = 2	24	137	1	1	5.63	0.02
A0A1L1RJ27	Fibroblast growth factor OS = *Gallus gallus* OX = 9031 GN = FGF5 PE = 3 SV = 2	21	27	1	1	11.6	0.12
A0A3G2WC03	Extracellular serine/threonine protein kinase FAM20C isoform X1 OS = *Gallus gallus* OX = 9031 PE = 2 SV = 1	18	61	1	1	9.11	0.05
**(b) List of VHH74-binding proteins from DCs identified by LC–MS**							
E1C1I6	Aldo_ket_red domain-containing protein OS = *Gallus gallus* OX = 9031 GN = LOC772271 PE = 3 SV = 2	59	36.1	2	2	6.8	0.19
A0A1D5NTT4	Voltage-dependent anion-selective channel protein 1 OS = *Gallus gallus* OX = 9031 GN = VDAC1 PE = 3 SV = 2	53	33.5	1	1	9.12	0.1
F1NS44	RING-type E3 ubiquitin transferase OS = *Gallus gallus* OX = 9031 GN = UHRF2 PE = 4 SV = 2	34	93.6	1	1	6.95	0.04
F1NSA8	Uncharacterized protein OS = *Gallus gallus* OX = 9031 GN = RAP1A PE = 1 SV = 2	33	21.3	1	1	6.4	0.16
A0A1D5P2Z8	Uncharacterized protein OS = *Gallus gallus* OX = 9031 GN = GPC2 PE = 3 SV = 2	29	65.2	1	1	6.23	0.05
A0A1D5P1A7	UBX domain-containing protein 2B OS = *Gallus gallus* OX = 9031 GN = UBXN2B PE = 4 SV = 2	28	44.9	1	1	8.57	0.07
A0A3Q2TWG3	Protein kinase domain-containing protein OS = *Gallus gallus* OX = 9031 PE = 4 SV = 1	27	39.1	1	1	4.91	0.08
P15988	Collagen alpha-2(VI) chain OS = *Gallus gallus* OX = 9031 GN = COL6A2 PE = 2 SV = 1	26	110.3	1	1	5.66	0.03
A0A1D5P5J3	Uncharacterized protein OS = *Gallus gallus* OX = 9031 GN = CDH15 PE = 4 SV = 1	25	86.01	1	1	5.04	0.04
F1NIE8	RanGAP1_C domain-containing protein OS = *Gallus gallus* OX = 9031 GN = RANGAP1 PE = 4 SV = 1	25	63.4	1	1	4.71	0.05
A0A1D5P5F1	zf-C2H2_7 domain-containing protein OS = *Gallus gallus* OX = 9031 GN = PRR35 PE = 4 SV = 3	24	96.4	1	1	6.11	0.03

Matches-sig: proteins that were significantly higher than the spectrum match threshold; Sequences-sig: peptides that were significantly higher than the threshold; pI: calculated isoelectric point; emPAI: exponentially modified protein abundance index.

### DC targeting induced high-level antibody

**Figure 6:**
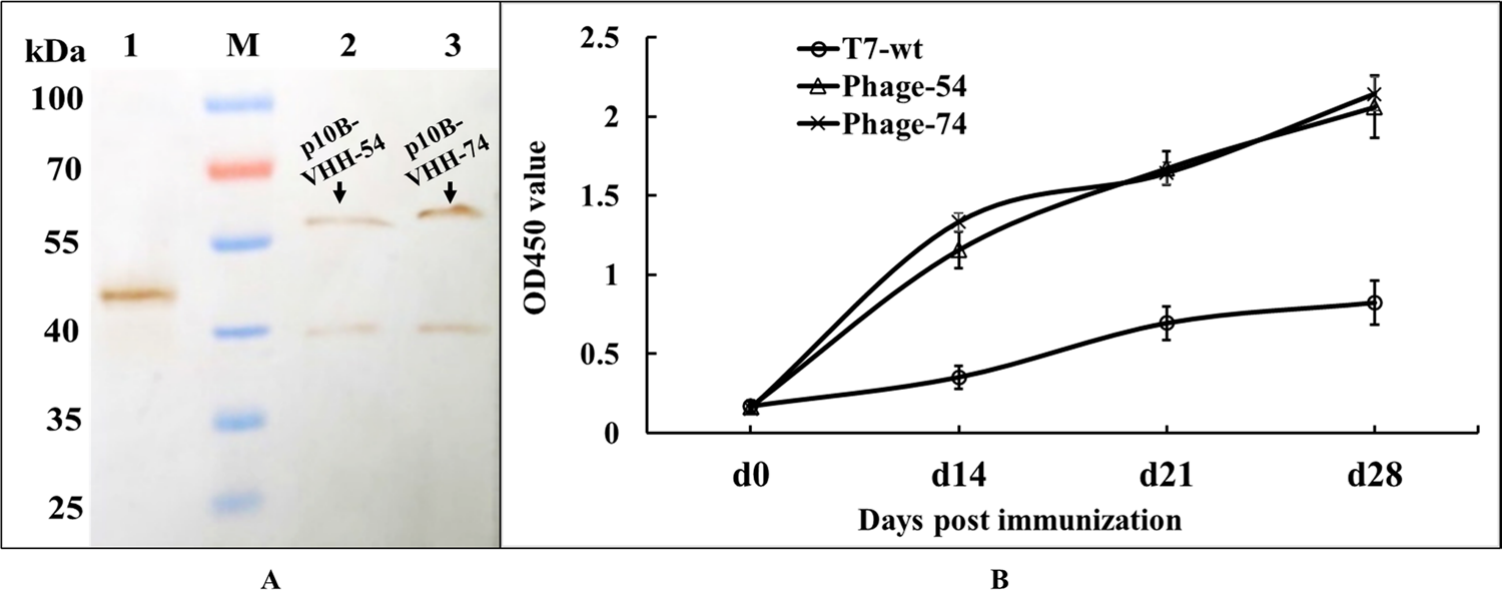
Detection of serum anti-p10B antibodies. (**A**) T7-wt, phage-54 and phage-74 were amplified, purified, and detected by Western-blot. Lane M: Pre-stained protein molecular weight marker (10 to 180 kDa, Fermentas), lane 1: T7-wt purified phage particles, band of p10B protein (38 kDa) with MCS was detected, lane 2: phage-54 purified phage particles, band of p10B-VHH-54 fusion protein (51 kDa) was detected, lane 3: phage-74 purified phage particles, band of p10B-VHH-74 fusion protein (51 kDa) was detected. (**B**) The detection of serum anti-p10B antibodies. Phage-54 and phage-74 were used as antigens to immunize SPF chickens and T7-wt was the negative control. Blood samples were collected from day 0 to day 28 post immunization. Purified T7 phage p10B proteins were coated for indirect ELISA detection of anti-p10B antibodies in the serum. Chickens immunized with phage-54 and phage-74 developed more rapid and higher levels of anti-p10B capsid antibody than chickens administered with the T7-wt control.

The purified T7-wt, phage-54 and phage-74 particles were detected by Western-blot and a single band of fusion protein p10B-VHH-54 and p10B-VHH-74 were obtained (Fig. 6A), which indicated a correct displaying of the nanobody on the T7 phage surface. To verify the function of DC-targeting nanobodies that act in the promotion of antigen presentation and antibody formation, groups of SPF chickens were subcutaneously injected with phage-54 and phage-74, with T7-wt as a control. The level of specific antibody against T7 phage capsid was determined using ELISA. T7 phage capsid was expressed in the *E. coli* system, purified (Supplementary Fig. S5) and used to coat ELISA plates to establish a detection method. Chickens immunized with phage-54 and phage-74 developed higher levels of anti-capsid antibody than chickens administered with the T7-wt control (Fig. 6B). DC- targeting phages were able to stimulate a more rapid and efficient immune response, thus indicating the potential application of the selected nanobody in antigen delivery.

## Discussion

In recent years, the use of DCs to improve the immunogenicity of an antigen has been a key strategy in the field of vaccine development^28,29^. These strategies can increase the number of antigens targeting DCs, and much progress has been made in human DC-targeting vaccines^30,31^. One of the common approaches to elicit a strong and long-lasting humoral and cellular immune response is the design of DC-targeting vaccines^32,33^. When researching and developing poultry vaccines, practical factors should be taken into consideration, including production cost and wide-scale administration, consequently, DC-targeting vaccines have become a desirable choice. Hence, we propose to discover chicken DC-specific binding ligands which could be used as an antigen carrier to develop DC-targeting vaccines.

Antibodies can specifically target receptors that are expressed on the surface of DCs, this concept has been utilized either by conjugating antigens to mAbs as DCs surface molecules, or by genetic engineering in which the antigen is fused to different antibody fragments specific to DC receptors^34^. Nanobodies, unique antibody-binding fragments derived from camelid heavy-chain antibodies, have excellent properties including thermal and chemical stability, weak immunogenicity and high affinity^35^. Thus, a T7 phage display nanobody library was constructed by inserting the alpaca VHH antibody gene into the downstream region of the T7 phage p10B gene, so that nanobodies could be displayed on the surface of the T7 phage. Compared to other phage display systems, the T7 select phage display system is easy to use and has the capacity to display peptides of about 50 to 1200 amino acids^36^. In this study, the high-copy number vector T7 select 415-1b was used to clone the VHH gene, and the *E. coli* BLT5403 bacterial host was used to supply the extra p10A protein to facilitate infectious recombinant phage rescue. Twenty plaques randomly screened by PCR, showed that the VHH gene was successfully inserted into the phage genome (Supplementary Fig. S1-A), and sequencing data revealed that the framework regions and complementarity determining regions of these VHH clones displayed the greatest difference in amino acid sequences (Supplementary Fig. S2). A small percentage of stop codons were detected within the VHH genes such as in VHH17 (Supplementary Fig. S2), which led to the truncated VHH protein bands being detected in the Western blot (Supplementary Fig. S1-B). In general, considering the high diversity of the VHH library and the efficient expression of VHH on the phage surface, this T7 phage display library could be adequate for bio-panning needs.

Antigen is bound with antibody and targeted to a DC receptor for internalization which can accelerate antigen processing and presentation^37^. The surface of DCs contains many pattern recognition receptors (PRRs). To date four PRRs have been found, including Toll-like receptors (TLRs), Nucleotide-binding oligomerization domain-like receptors (NLRs), Retinoic acid-inducible gene I-like helicases receptors (RLRs) and C-type lectin receptors (CLRs)^38,39^. Choosing the receptor to be targeted is a great challenge in the design of an antibody-based DC targeting vaccine. Although, these receptors combine specifically with the corresponding natural ligands, it is important to explore new ligands for binding with the reported receptors or other unknown receptors. For these reasons, the constructed nanobody library was used to screen intact chicken DCs, with the expectation of discovering DC-specific binding nanobodies. Although there was obvious enrichment after three rounds of screening (Fig. 3A), a decrease in phages with unique sequences was observed (Fig. 3B). Repeat phage clones were identified by sequence analysis (Supplementary Fig. S3) and this high frequency of repetition phage clones points to the success and enrichment of the screening process. As a result, twelve DC-specific binding phage clones were obtained (Fig. 3C). Unexpectedly, four of the selected phage clones bound not only to chicken DCs but also to duck and goose DCs (Fig. 4B–E). These results indicated that the chicken, duck and goose may share the common receptors that were recognized by these selected phages.

Currently, there is little consensus as to which receptor elicits more robust MHC I or MHC II antigen presentation^40^. Effective antigen presentation results from the antigen being trafficked to subcellular compartments for processing, however, individual DC receptors will differ widely in their expression levels, internalization speeds, and downstream intracellular trafficking pathways. In any event, the efficient combination of antigen and DCs is the first step for antigen processing. The interaction between the selected phage and DCs was studied by confocal laser microscopy and revealed that phage-54 and phage-74 could efficiently combine with chicken DCs within 15 min (Fig. 5). Further, nanobody binding proteins on DCs were obtained by pull-down assay and identified by mass spectrometry (Table 1). Unfortunately, none of the previously reported receptors were discovered during the mass spectrometry analysis. However, one protein, i.e., voltage-dependent anion-selective channel protein (VDAC), suspected to have a role in antigen processing was discovered. Lisanti et al.^41^ have previously reported the presence of VDAC1 in a catalogue of proteins identified in caveolae. Caveolae are domains of the plasma membrane that have specific functions in the trafficking between the plasma membrane and the rest of the cell^42^. Thus, it may be presumed that the specific binding of phage-74 to the VDAC of DCs made the engulfment easier, and further, sped up processing and presentation of the antigen. This supports the more rapid and higher level of the antibody response against p10B elicited by the DC-targeting nanobodies of phage-54 and phage-74 compared to that induced by T7-wt (Fig. 6B).

## Conclusion

In this study, a high diversity T7 phage display nanobody library was constructed which could be used for bio-panning of chicken DC-specific binding nanobodies. The results indicated that nanobodies displayed on phage-54 and phage-74 not only efficiently bind with chicken DCs but also to duck and goose DCs. Although the exact nanobody recognition receptor requires further elucidation, the highly efficient affinity binding of nanobody to DCs promotes the immune response. Therefore, the nanobody displayed on phage-54 and phage-74 is a DC-targeting ligand that merits further study and application.

## Methods

### Ethics declarations

Animals were maintained and euthanized as per the protocol, approved by the Institutional Animal Care and Use Committee (IACUC) of the Jiangsu Academy of Agriculture Sciences (SYXK 2017–2022). All experiments were conducted in accordance with the relevant guidelines and regulations of IACUC and the Institutional Biosafety Committee at the Jiangsu Academy of Agriculture Sciences. This study is reported in accordance with ARRIVE guidelines (https://arriveguidelines.org).

#### Construction of T7 phage display nanobody library

A T7 phage display nanobody library was constructed as previously described^43^. The nanobody was displayed as an extension of the coat protein due to an in-frame insertion of the alpaca VHH gene in the p10 gene encoding the coat protein of T7 phage, resulting in the display of less than 450 guest nanobodies on the surface of each phage particle. Briefly, anticoagulated blood samples were collected from six non-immunized young alpacas (three female and three male) and lymphocytes were isolated using the Ficol separation method^44^. Total RNA was extracted using the MiniBEST Universal RNA Extration Kit (TaKaRa) and then first stand cDNA was generated using PrimeScript 1st Strand cDNA Synthesis Kit (TaKaRa). The VHH gene was amplified by the stepwise PCR method^45^ and all the primers used are presented in Table S1. The VHH gene products were digested with restriction enzymes EcoRI and HindIII and ligated to the T7 select 415-1b EcoRI/HindIII vector arms (Merck, Germany) to rescue the primary phage display library. This primary T7-VHH library was amplified using the liquid lysate amplication method according to manufacturer’s instructions. The titers of the primary and amplified library were determined by phage-plaque assay using *Escherichia coli* BL5403 host^46^. Nanobody displayed on T7 phage particles were detected by SDS-PAGE and Western-blot as previously reported^47^.

#### Isolation and validation of chicken bone marrow DCs

Marrow collected from the femurs and tibias of three week-old specified pathogen-free (SPF) chicks was washed three times with sterile phosphate buffered saline (PBS), gently loaded in an equal volume of Histopaque-119 (Sigma, Germany), then centifuged at 250 × *g* for 30 min. Cells at the interface were collected and washed twice with PBS as previously described^48^. Aliquots of 2 × 10^6^ cells/mL were used to seed 6-well plates containing Roswell Park Memorial Institute (RPMI) 1640 medium supplemented with 1 U/mL penicillin and streptomycin, 10% fetal bovine serum (FBS, Gibco, USA), 30 ng/mL recombinant human granulocyte macrophage-colony stimulating factor (GM-CSF) and 25 ng/mL interleukin-4 (IL-4) (Peprotech) and incubated at 37 °C and 5% CO_2_ for 6 d. Fifty percent of the medium was replaced with complete medium every two days. Cytomorphology of DCs was observed under an optic microscope during the cell differentiation process. CD11c, CD86 and MHCII expressed on the surface of DCs on the 6th day were analyzed using fluorescence-activated cell sorting (FACS) (BD FACSCalibur, FACS101) at Taizhou People’s Hospital. The duck and goose bone marrow DCs were prepared similarly.

#### Bio-panning of DC-specific binding phages

The T7-VHH library displaying alpaca nanobody at the C-terminal of p10B protein was applied to screen the DC-specific binding nanobody^10^. Depletion selection: monocytes isolated from bone marrow were resuspended in RPMI 1640 medium supplemented with 10% FBS and the density adjusted to 1 × 10^7^ cells/mL. An aliquot of the T7-VHH library containing ~ 10^10^ PFU phages was diluted in blocking buffer (RPMI 1640 medium supplemented with 10% FBS + 0.5% bovine serum albumin) and transferred to 6-well plates and left for 1 h at room temperature to deplete the library of phages that adsorb to the plastic. Unbound phages were removed and tranferred to another 6-well plate containing monocytes, then incubated for one hour at room temperature to deplete monocyte-binding phages. First round: the supernatants of the monocytes plate were transferred after centrifugation (250×*g* for 5 min) to incubate with DCs in a 6-well plate at room temperature for 45 min. The plate was then centrifuged at 250×*g* for 5 min, supernatants were removed, and cells resuspended in wash buffer (RPMI 1640 medium containing 1% FBS and 0.05% Tween-20). Washes were collected and saved for titering the phage. The wash operation was repeated five times. Thereafter, the DC-bound phages were eluted by lysing the cells with CHAPS lysis buffer (2.5% *w/v* CHAPS [3-((3-cholamidopropyl) dimethylammonio)-1-propanesulfonated] in RPMI 1640 medium) and evaluated by the phage-plaque assay^46^. The phages were amplified in *E. coli* BL21 for the next round of bio-panning. The second and third rounds were carried out according to the procedures described above, except that the incubation time was reduced to 30 min and 15 min, respectively. The VHH genes in the total eluted phage from each round of selection were amplified, and the PCR products were sent to Genepioneer Biotechnologies Co., Ltd. (Nanjing, China) for next-generation sequencing. The individual phage plaques in the eluate of the third round of selection were randomly selected for VHH gene amplification^36^, and PCR products were sequenced by Genscript Biotechnology Co., Ltd. (Nanjing, China).

#### Specificity and selectivity assay

Individual phage clones identified by DNA sequencing were propagated and purified^26^ for use in cell-association assays. In the specificity assay, phage particles (~ 10^6^ PFU/ well) were incubated with DCs, chicken bone marrow cells and serum-treated control wells in a 96-well cell culture plate at room temperature for 15 min. Following several washes, cell- or serum-associated phages were collected by treating each well with CHAPS lysis buffer and titering in *E. coli* BL5403 cells. The most promising phage binders, i.e., those phages that demonstrated increased binding to DCs rather than bone marrow cells and serum components, were tested further for their ability to discriminate between different targets (selectivity assay) using a panel of different cells from chicken, duck and goose. Dendritic cells and bone marrow cells of chicken, duck and goose were prepared as previously mentioned. Chicken embryo fibroblasts (CEF), duck embryo fibroblasts (DEF) and goose embryo fibroblasts (GEF) were prepared according to the method of Zhai et al.^49^. Cells were seeded into 96-well culture plate (~ 10^4^ cell/well) in a 37 °C cell culture incubator with 5% CO_2_ for 1 h. Then, each phage clone (~ 10^6^ PFU/well) was incubated with cells for 15 min at room temperature. Wells were washed eight times with washing buffer by centrifugation of plate at 250×*g* for 5 min and unbound phages in supernatant were carefully removed. To collect cell-associated phages, 25 μL of CHAPS lysis buffer was added to each well and incubated for 10 min on a shaker with gentle rocking. Aliquots of 175 μL of overnight cultured *E. coli* BL5403 host cells were added to the each well and incubated for 3 min at room temperature. The final mixture was spread on LB agar plates and incubated for 3–4 h in a 37 °C incubator. The phage recovery was calculated as the percent ratio of output plaque forming units to input plaque forming units. All selectivity and specificity cell-associated assays were performed in triplicate with data reported as the mean ± standard deviation.

#### Subcellular localization assay

Interactions of selected phage clones with DCs were analyzed as described previously^50^. Dendritic cells at day 6 were harvested and seeded on a cell slide, and then incubated in a 37 °C incubator with 5% CO_2_ until cells were ~ 70% confluent. Next, cells were incubated with 1.0 × 10^9^ PFU phages of an isolated phage clone in serum-free RPMI 1640 culture medium for 15 min at 37 °C. Cells were washed five times with PBS and fixed with 4% paraformaldehyde for 20 min at room temperature. After an additional three washes, cells were permeabilized with 0.1% Triton X-100 for 10 min and blocked with 1% bovine serum albumin for 30 min at room temperature. Cells were treated with a 1:2000 dilution of Dylight Anti-T7 tag antibody (ab117595, abcam) in blocking buffer for 1 h at room temperature. Cells were washed with PBS containing 0.05% Tween-20 (PBST) and treated with a 1:1000 dilution of Phalloidin-iFluor 594 Conjugate (abs42235791, absin) for 1 h at room temperature in the dark. After washing, cover slips were applied to the slides with VECTA shield mounting medium and DAPI. Slides were visualized with a ZEISS confocal microscope (ZEISS LSM 880) at the Testing & Analysis Center at Yangzhou University. The VHH genes from phage-54 (VVH-54) and phage-74 (VVH-74) were fused with the glutathione S-transferase (GST) gene in pGEX-4T-1 vector for fusion protein expression. The purified fusion protein and immature chicken DCs (day 6) were sent to ZoonBio Biotechnology company (Nanjing, China) for in vitro pull-down and mass spectrometry assays.

#### Immunogenicity of T7 phage displaying DCs nanobody

The T7-wt (T7 select 415-1b), phage-54 and phage-74 were propagated in *E. coli* BL5403. Briefly, 50 mL of LB medium was inoculated with 500 μL of an over-night cultured *E. coli* BL21 and incubated with shaking (200 rpm, 2.5 h) at 37 °C to reach a density at *OD*_600 nm_ of 1.0. The *E. coli* BL5403 host was then infected with the phage particles at a multiplicity of infection (MOI) of 0.001 and kept shaking at 37 °C for more than 3 h until complete cell lysis was observed. DNase I and RNase A (Takara, China) were added 30 min before harvesting the offspring phages. Phage particles were recycled by the PEG-NaCl method and extracted with 0.1% Triton-X114 to remove endotoxin^26^. The purified T7-wt, phage 54 and phage 74 were detected by Western-blot, then the titer of phages was adjusted to 10^11^ PFU/mL and inactivated by 0.1% (v/v) β-propiolactone. Further, the ratio of aqueous phage to the Montanide ISA 206 (Seppic France) oil adjuvant was 54:46 (V/V) to form a water-in-oil-in-water (W/O/W) blend. Thirty SPF chickens were divided into three groups, and their necks injected subcutaneously with 0.2 mL of T7-wt, phage-54 and phage-74 emulsion, respectively. Blood samples were collected after days 0, 14, 21 and 28, and T7 phage anti-capsid antibody levels were measured using ELISA. The capsid protein of T7 phage (p10B) was expressed by pET-28a-p10B vector, and the purified protein (100 ng/mL) was used for coating to establish an indirect ELISA method. A more detailed description about the ELISA protocol used is provided in the supplementary material. All chickens were maintained and euthanized as per the protocol approved by the Institutional Animal Care and Use Committee and conducted following the guidelines of the Institutional Biosafety Committee at the Jiangsu Academy of Agriculture Sciences.

## Supplementary Information

Supplementary Information.

## Data Availability

Supplementary information accompanies this paper.
