# Supplementary material for: Construction of a T7 phage display nanobody library for bio-panning and identification of chicken dendritic cell-specific binding nanobodies
Source: Sci Rep. 2022 Jul 15;12:12122. doi: 10.1038/s41598-022-16378-x (PMC9284966; doi:10.1038/s41598-022-16378-x)
Supplement: Supplementary file 1 — Supplementary Information. [file 41598_2022_16378_MOESM1_ESM.pdf]

Supplementary materials for

Construction of a T7 phage display nanobody library for bio-panning and identification of chicken dendritic cell-specific binding nanobodies

Hai Xu [1,2,3], Ling Li [1], Bihua Deng [3,4*], Weiming Hong [1], Ruiting Li [1], Zijie Guo [1], Jibo Hou [3], Roshini Govinden [2], Hafizah Y. Chenia [2,*]

This file includes:

ELISA method

Figures S1 to S5

Table S1

Original gel and Western blot images

Establishment of an indirect ELISA method for detection of antibody to T7 phage

An aliquot of 100 ng/ml of purified p10B protein was immobilized on the wells of the plate (Corning) at 4°C overnight. Wells were washed with PBS and blocked with 1% BSA in PBS for 2 h at 37°C. Aliquots of 100 µl of hundredfold diluted serum samples were then added to each well and incubated for 1 h at 37°C. The wells were then washed with PBS containing 0.1% tween 20 (PBST) before the addition of a 100 µl PBS containing goat anti-chicken IgY antibody conjugated with horseradish peroxidase (1:10000, Abcam) and 5% nonfat dry milk. After a further incubation of the plates at 37°C for 1 h, wells were washed with PBST. The color reaction at 37°C for 15 min was initiated by the addition of 100 µl solution containing 0.4 mg/ml 3,3’,5,5’-tetramethylbendizine and 0.015% H_2O_2. The reaction was terminated by the addition of 50 µl of 1 M H_2SO_4, and the absorbance at 450 $_{nm}$ was measured with a microplate reader.

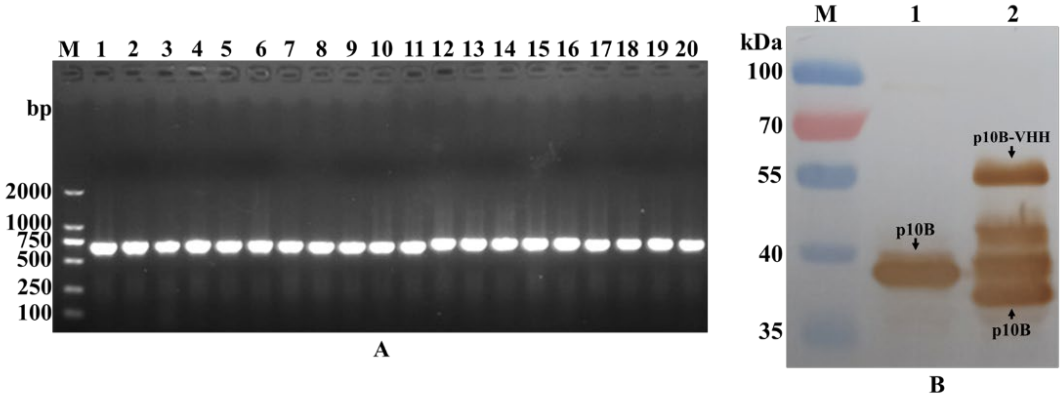

FIGURE S1 Identification of T7-VHH library. (A) Plaque PCR detection of single clone phage. Lane M: DL2000 marker (100 to 2000 bp, Takara) and lanes 1-20: Twenty randomly selected phage plaques for PCR detection. (B) Western-blot analysis of nanobody displayed on the T7 phage. Lane M: Pre-stained protein molecular weight marker (10 to 180 kDa, Fermentas), lane 1: p10B protein (38 kDa) of T7 selected 415-1b (T7-wt) fused with MCS and lane 2: Four bands were detected in the phage library (T7-VHH) including p10B-VHH fusion protein (51 kDa), p10B protein (36 kDa) expressed by the host bacterium *E. coli* BL5405 without a MCS and two expressed but truncated p10B-VHH fusion proteins.

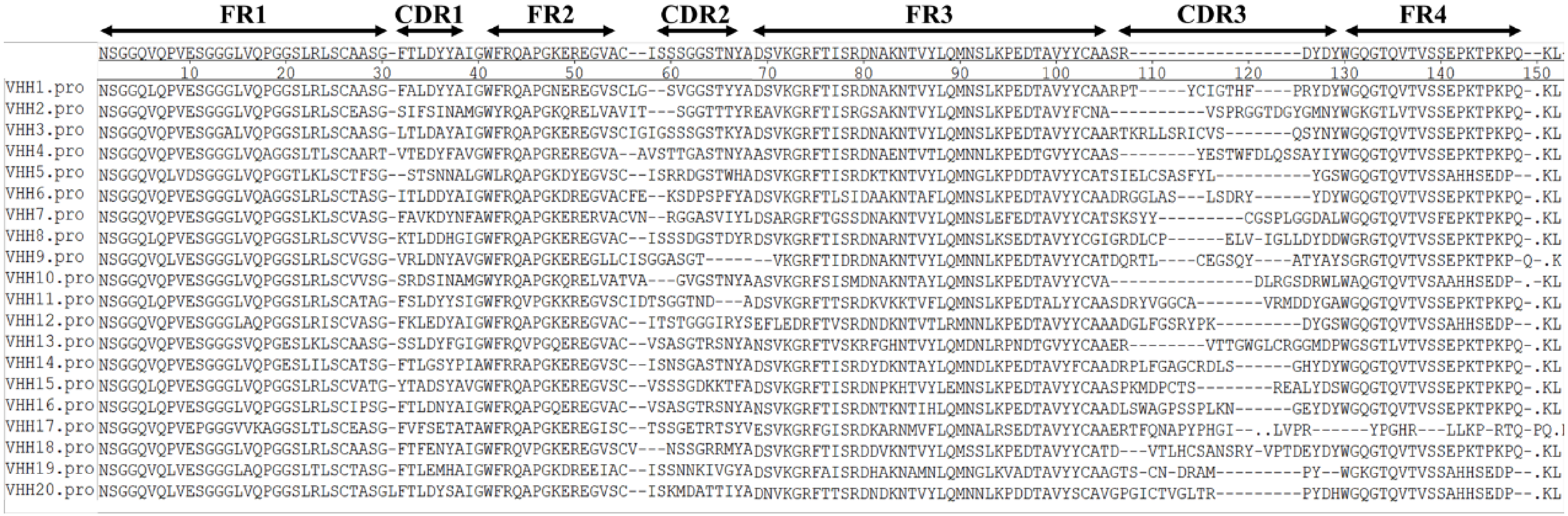

FIGURE S2 Sequencing analysis of T7-VHH library. PCR products of 20 randomly selected phage plaques were sequenced and the coding proteins were analyzed. Sequence organization of the VHH clones is depicted with four frameworks (FW1-FW4) and three complementary determining regions (CDR1-CDR3). The amino composition of FW region was relatively conserved while high diversity of the amino composition was observed in the CDRs. A stop codon was discovered in the CDR3 of VHH17 which resulted in expression of a truncated p10B-VHH protein.

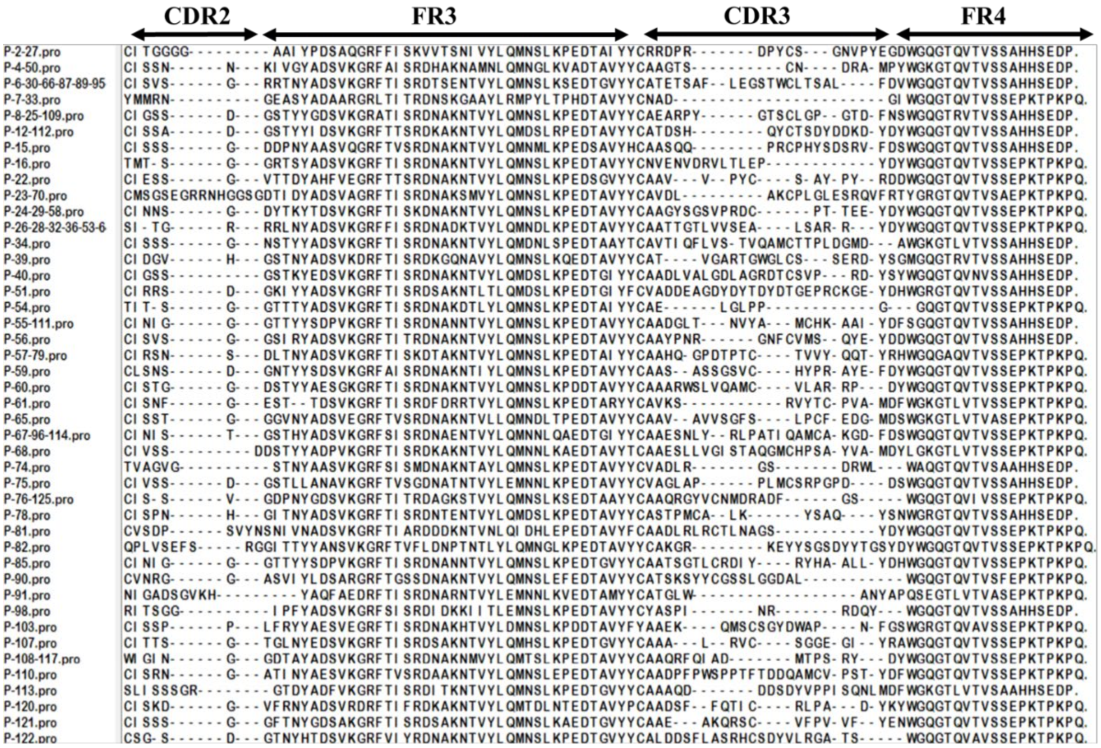

FIGURE S3 Sequencing analysis of unique phage clone from the 3rd round of bio-panning. After three rounds of screening, 125 phage clones were randomly selected for sequencing, and 46 phage clones with unique complementary determining region (CDR) sequence were identified. Partial sequence of the identified phage clones is depicted with FR3, FR4 and CDR2, CDR3. The amino composition of the CDR was different between each clone and no stop codons were discovered.

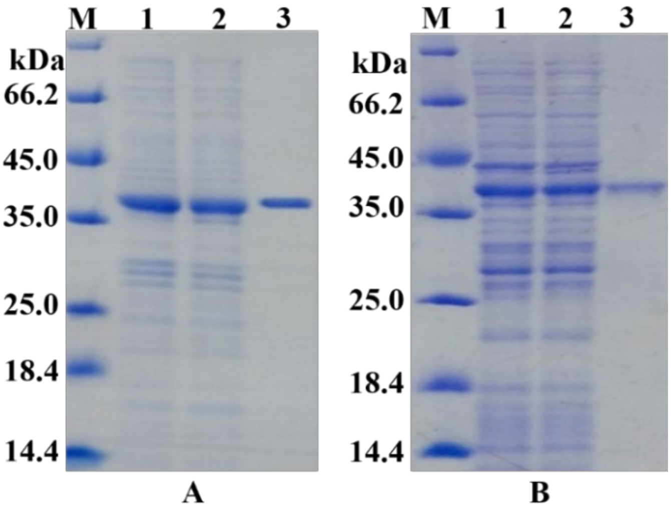

FIGURE S4 The expression and purification of GST-fused nanobody. (A) The fusion protein GST-VHH54 was expressed using the pGEX-4T-VHH54 vector. Lane M: Protein molecular weight marker (14.4 to 116.0 kDa, Fermentas), lane 1: IPTG-induced protein samples inserted into GST resin column, lane 2: Protein samples in the flow through from the GST resin column and lane 3: Purified GST-VHH54 protein eluted from the column. (B) Fusion protein GST-VHH74 was expressed using the pGEX-4T-VHH74 vector. Lane M: Protein molecular weight marker (14.4 to 116.0 kDa, Fermentas), lane 1: IPTG-induced protein samples inserted into GST resin column, lane 2: Protein samples in the flow through from GST resin column and lane 3: Purified GST-VHH74 protein eluted from the column.

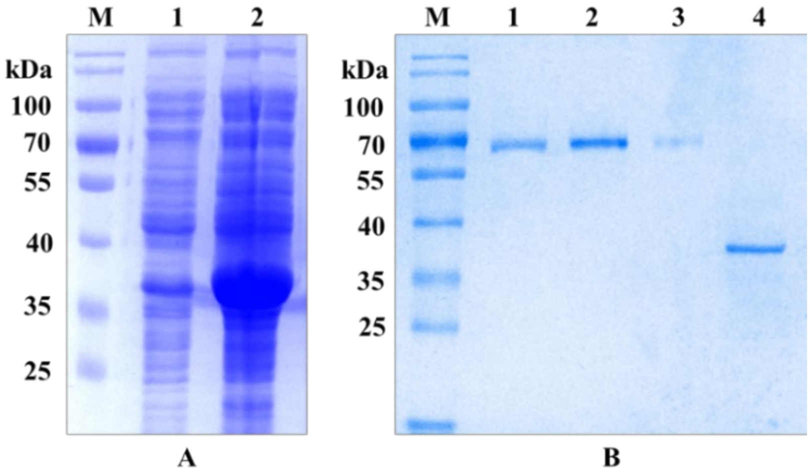

FIGURE S5 The expression and purification of the p10B protein. (A) Protein p10B of T7 phage was expressed using the pET-28a-p10B vector. Lane M: Pre-stained protein molecular weight marker (10 to 180 kDa, Fermentas), lane 1: *E. coli* BL21-DE3 host cell and lane 2: IPTG induced *E. coli* BL21-DE3 host cell containing the pET-28a-p10B vector. (B) Lane M: Prestained protein molecular weight marker (10 to 180 kDa, Fermentas) and lanes 1-3: BSA reference standard and 4: His tag purified p10B protein.

Table S1

Primers used in library construction and identification

Primer	Primer sequence（5’→3’）
F1	CTGGGTGGTCCTGGCTGCTCTT
R1	GCGGTACGTGTGTTGAACTGTT
F2	GAATTCGGGTGGTCAGKTGCAGCYCGTGGAGNC
R2-1	AAGCTTTTATTGTGGTTTTGGTGTCTTGGG
R2-2	AAGCTTTTAGGGGTCTTCGCTGTGGTGCG
T7 select up	GGAGCTGTCGTATTCCAGTC
T7 select down	AACCCCTCAAGACCCGTTTA

Note: K=G, T; Y=C, T; N=A, G, C, T. The enzyme digestion sites are underlined.

Original gels and Western blots

Original blot for Figure 6-A

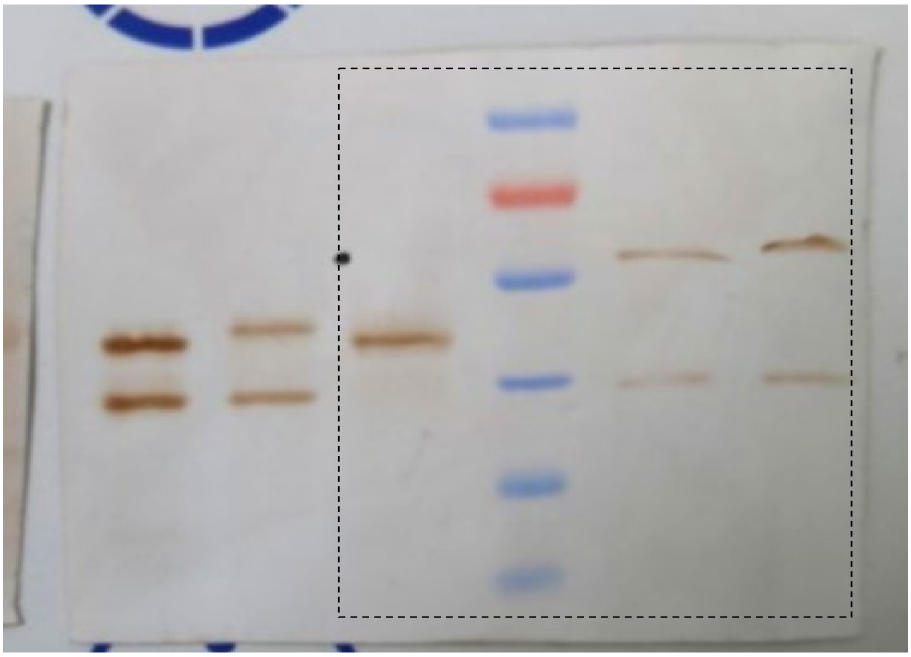

The original blot image of Figure 6-A. The blot in dotted-box was used in the manuscript.

Original blot of Figure S1-B

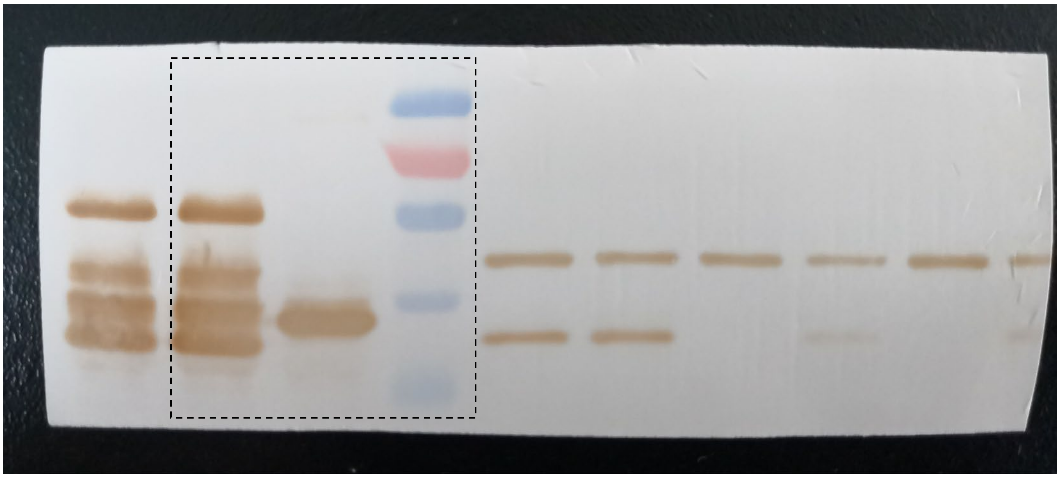

The original blot image of Figure S1-B. The blots in dotted-box were used in supplementary materials. Image was inverted for the manuscript so that marker was on left hand side.

Supplementary Figure 4-A

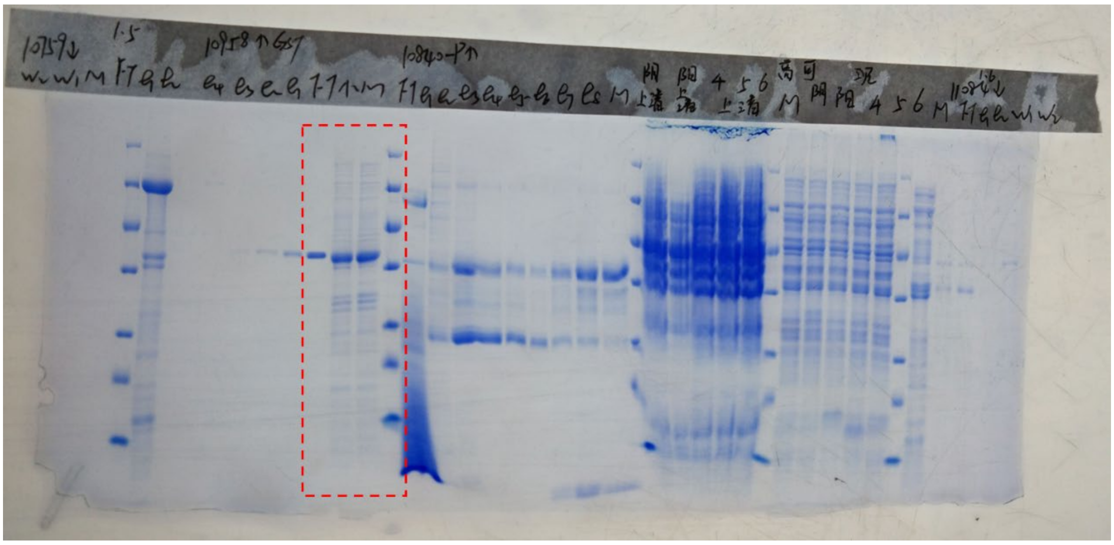

Supplementary Figure 4-B

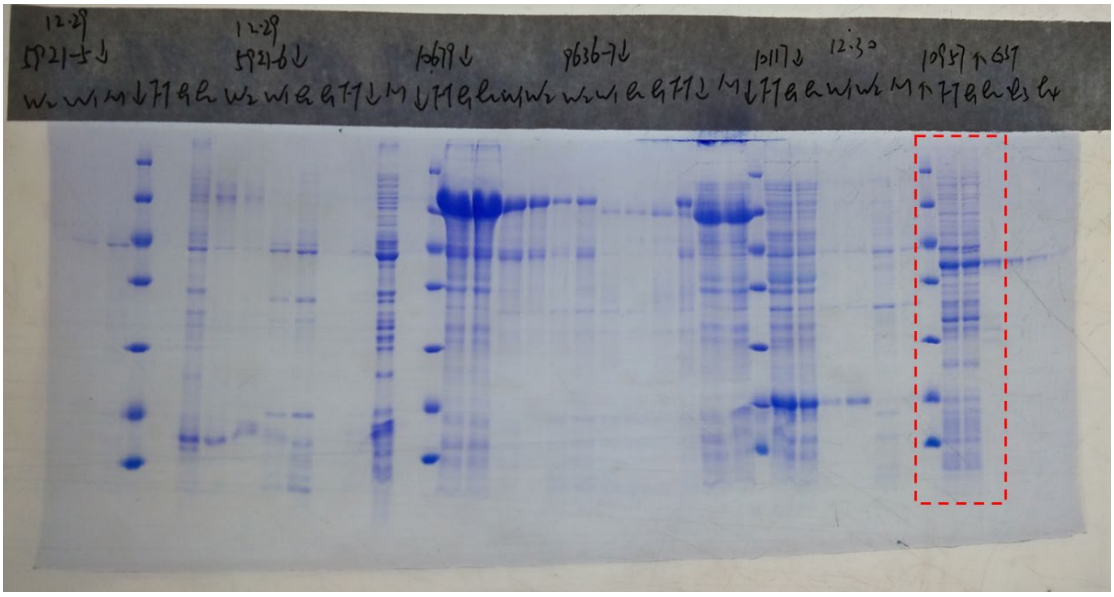

The original acrylamide gel images of Figures S4-A and S4-B. The lanes in dotted-box were used in supplementary materials.

Supplementary Figure 5-A

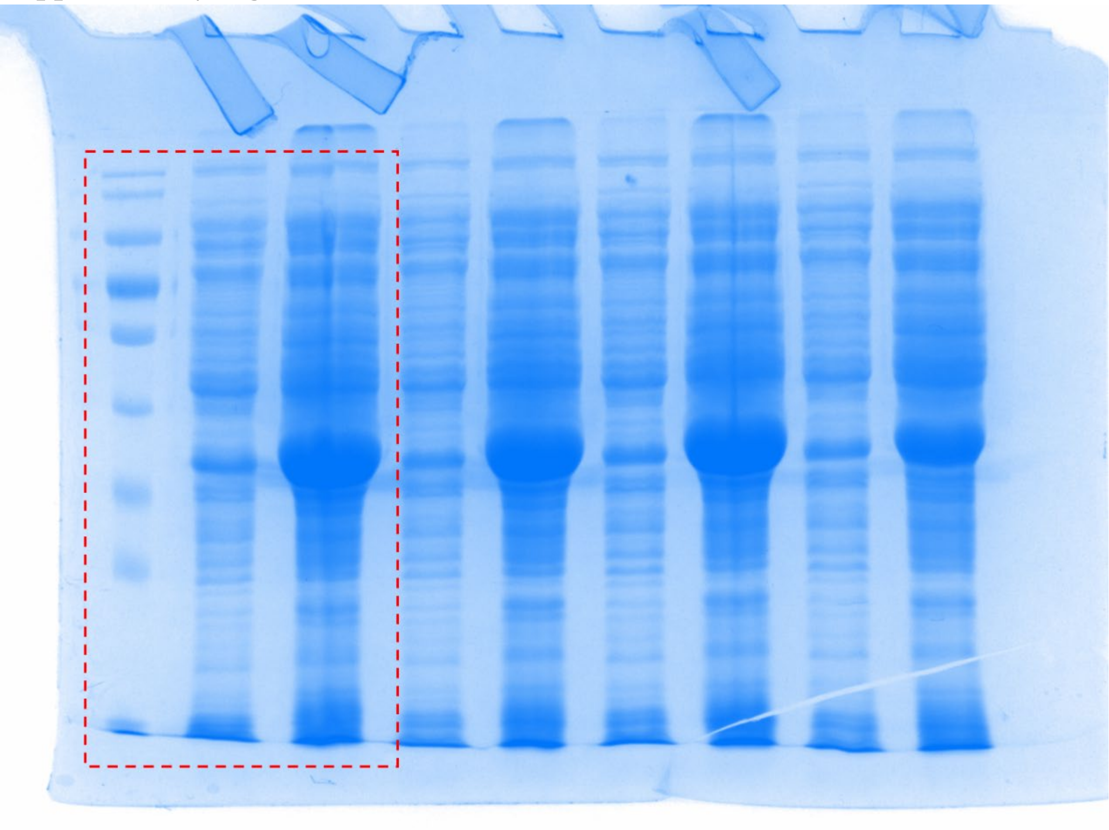

Supplementary Figure 5-B

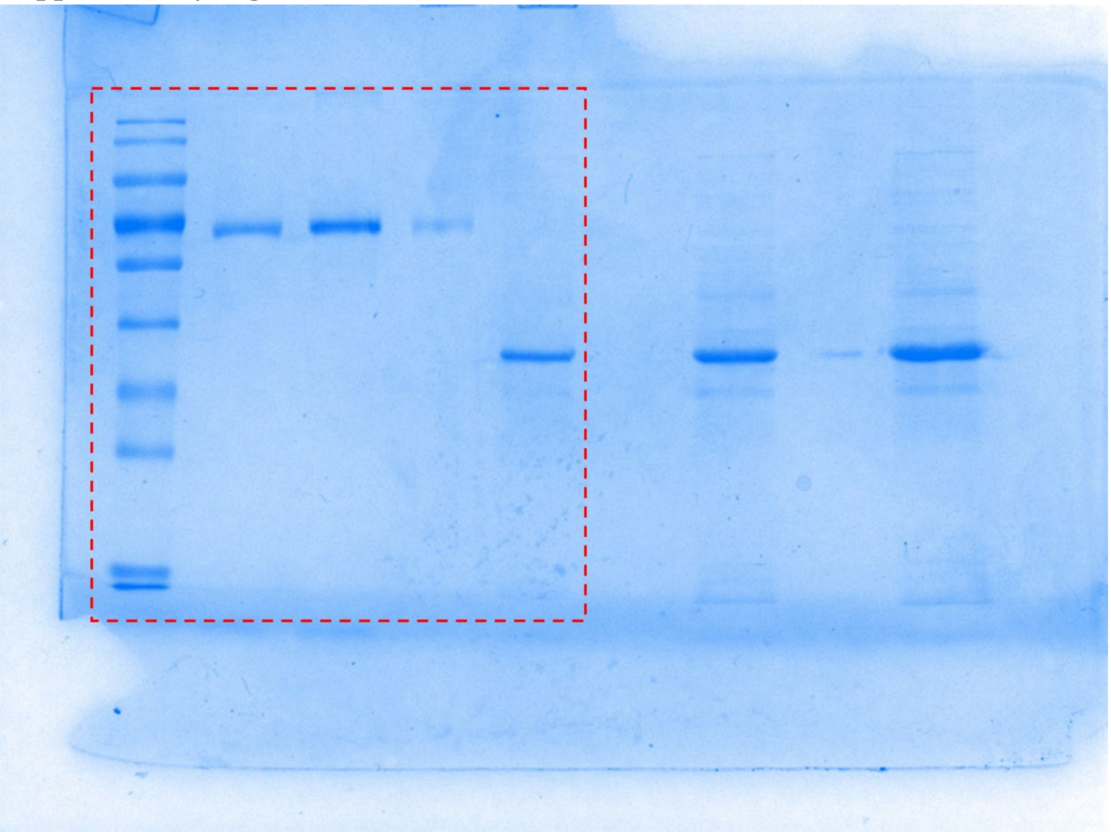

The original acrylamide gel images of Figures S5-A and S5-B. The lanes in dotted-box were used in supplementary materials.
